# “Distribution of dominant wavelengths predicts jackdaw (*Corvus monedula*) color discrimination performance”

**DOI:** 10.3389/fphys.2025.1543469

**Published:** 2025-02-20

**Authors:** Farina Lingstädt, Aylin Apostel, Jonas Rose

**Affiliations:** Neural Basis of Learning, Department of Psychology, Ruhr University Bochum, Bochum, Germany

**Keywords:** color discrimination, jackdaws (*Corvus monedula*), color vision, psychophysics, dominant wavelength, color stimuli

## Abstract

Color vision is an important perceptual ability in most species and a crucial capacity underlying any cognitive task working with color stimuli. Birds are known for their outstanding vision and tetrachromacy. Two jackdaws were trained to indicate whether they perceive two colors as same or different. The dominant wavelengths of the experimental colors were assessed to relate the birds’ performance to the physical qualities of the stimuli. The results indicate that the differences or similarities in dominant wavelengths of the colors had a strong influence on the behavioral data. Colors related to a reduced discriminatory performance were colors of particularly close wavelengths, whereas differences in saturation or brightness were less relevant. Overall, jackdaws mostly relied on hue to discriminate color pairs, and their behavior strongly reflected the physical composition of the color set. These findings show that when working with color stimuli, not only the perceptual abilities of the particular species, but also the technical aspects concerning the color presentation have to be considered carefully.

## Introduction

Birds are highly visual animals (even caricatured as “eyes with wings”, see Jones et al., 2007; Martin, 2012) and possess a complex visual system comprising not only three (as in humans and some other primate species; Jacobs, 2009), but four distinct photoreceptor pigments, which in some bird species has already been shown to enable tetrachromatic vision (Kelber et al., 2003). In modern cognitive neuroscience, various avian species are used in a wide range of studies probing cognitive abilities such as working memory (e.g., Diekamp et al., 2002 with pigeons and Hahn et al., 2021 with crows), categorization (e.g., Apostel et al., 2023a with jackdaws and Wasserman et al., 2023 with pigeons), and numerosity (e.g., Kobylkov et al., 2022 with chicks and Wagener et al., 2018 with crows). Almost all these studies use colorful visual stimuli displayed on computer monitors. For us as humans, this represents a clear and vivid illustration of the experimental approach. However, it is fundamental to also consider the specific visual capabilities of each bird species and general differences from trichromats (Hart and Hunt, 2007). We propose that objective measures of physical stimulus properties and psychophysical assessments of color discrimination are needed to more accurately interpret and understand experimental results in the work with avian subjects.

Corvids in particular show remarkable abilities in higher cognitive functions, such as working memory (Hahn et al., 2021; Veit et al., 2014), categorization (Apostel et al., 2023a; Vernouillet et al., 2021), rule-guided behavior (Veit and Nieder, 2013; Wilson et al., 1985), episodic-like memory (Clayton and Dickinson, 1998; Emery and Clayton, 2004), and tool use (Rutz and St. Clair, 2013; Rutz et al., 2016). Despite their focus on higher cognition, studies researching cognitive abilities such as working memory capacity also need to consider the perceptual abilities of their research subjects. This is especially relevant for studies targeting working memory, since change-detection paradigms use color among the items’ properties to probe capacity limitations (e.g., Balakhonov and Rose, 2017). A recent study on working memory in jackdaws, members of the corvid family, investigated the effects of enhanced memory demands on the accuracy of color working memory representations (Apostel et al., 2023b). Not only have the authors demonstrated similar biases towards categorical representations of color between corvids and primates, but they also discussed differences in color perception and the importance of avian calibrated experimental conditions. We intend to build up on this discussion, by assessing the color discrimination performance of jackdaws and its relationship to the physical properties of the experimental colors.

It is well established that the visual spectrum of several bird species includes ultraviolet light (Hart and Hunt, 2007; Kelber, 2019). However, an assessment of how this influences the perception of the remaining colors is mostly lacking (with the exception of hummingbird color vision, see Herrera et al., 2008; Stoddard et al., 2020). So far, only few attempts of an actual psychometric assessment of color discrimination abilities in birds were made that span only a small range of species (see Wright, 1972 for pigeons; Stoddard et al., 2020 for hummingbirds and Olsson et al., 2015 for chickens). The results obtained so far make it apparent that psychometric functions of color discrimination obtained in one bird species are not necessarily transferable to others. Additionally, avian species most used in higher cognitive tasks, such as parrots and corvids, have not been properly assessed in this regard. In order to accurately assess the performance of corvids in cognitive tasks involving colored stimulation, the current study investigated the color discrimination abilities of jackdaws using methods of color generation and presentation that were also used in other studies (see, e.g., Apostel et al., 2023b). To better interpret the performance of the birds with respect to the specific stimulus set, the main physical characteristics of the colors, dominant wavelength, luminance, and saturation, were assessed and correlated with the discrimination curve obtained from the behavioral data.

We found that the performance of the birds in the color discrimination task was mostly dependent on hue, with luminance and saturation only accounting for a small part of the variance in the data. Additionally, we confirmed previously reported challenges in color display (Bae et al., 2014) – color stimuli created to be isoluminant and with equidistant hues deviated significantly from the actual colors rendered on a PC screen, even though this screen was specifically chosen for its distinctive color rendering and display quality. This study highlights both the need for species-specific evaluations of color discrimination as well as thorough assessments of the physical characteristics of the color stimuli to better interpret experimental data.

## Methods

### Subjects

The subjects of this study were two jackdaws, approximately 3.5 years of age. They came from the same hand-raised colony and were thus exposed to the same environment while growing up. The subjects were randomly chosen from a larger social group housed in a spacious indoor aviary with a 12-h day and night cycle and unrestricted access to water and grit. During the behavioral training and experimental sessions, access to food was restricted according to a food protocol that allowed food-pellets (BEO special, Vitrakraft) to be used as reward. On days without training or testing, food was given *ad libitum*. All experimental procedures and housing conditions were carried out in accordance with the National Institutes of Health Guide for Care and Use of Laboratory Animals and were authorized by the national authority (LANUV).

### Behavioral setup

The behavioral experiments were performed in a Skinner box with a size of 71 cm (width), by 48 cm (depth) by 80 cm (height). The box was equipped with a 27″ monitor (AW2720HF, dell Alienware) and a 29 cm (width) by 49 cm (height) infrared touch frame (PQ Labs G4). The experiments were conducted in completely dark conditions within the Skinner box, illuminated only by the visual display from the monitor. The birds were positioned on a perch 14 cm away from the monitor, their behavior was remotely monitored with an IP camera (Edimax), and correct responses were rewarded with a custom-made automatic pellet feeder (https://www.ngl.psy.ruhr-uni-bochum.de/ngl/shareware/pellet-feeder.html.en). All experiments were controlled by custom MATLAB code, using the OTBR (Otto and Rose, 2023) and Psychophysics (Brainard and Vision, 1997) toolboxes. The digital input and output to and from the controlling PC were handled by a custom-built microcontroller (ODROID).

### Color stimuli

**FIGURE 1:**
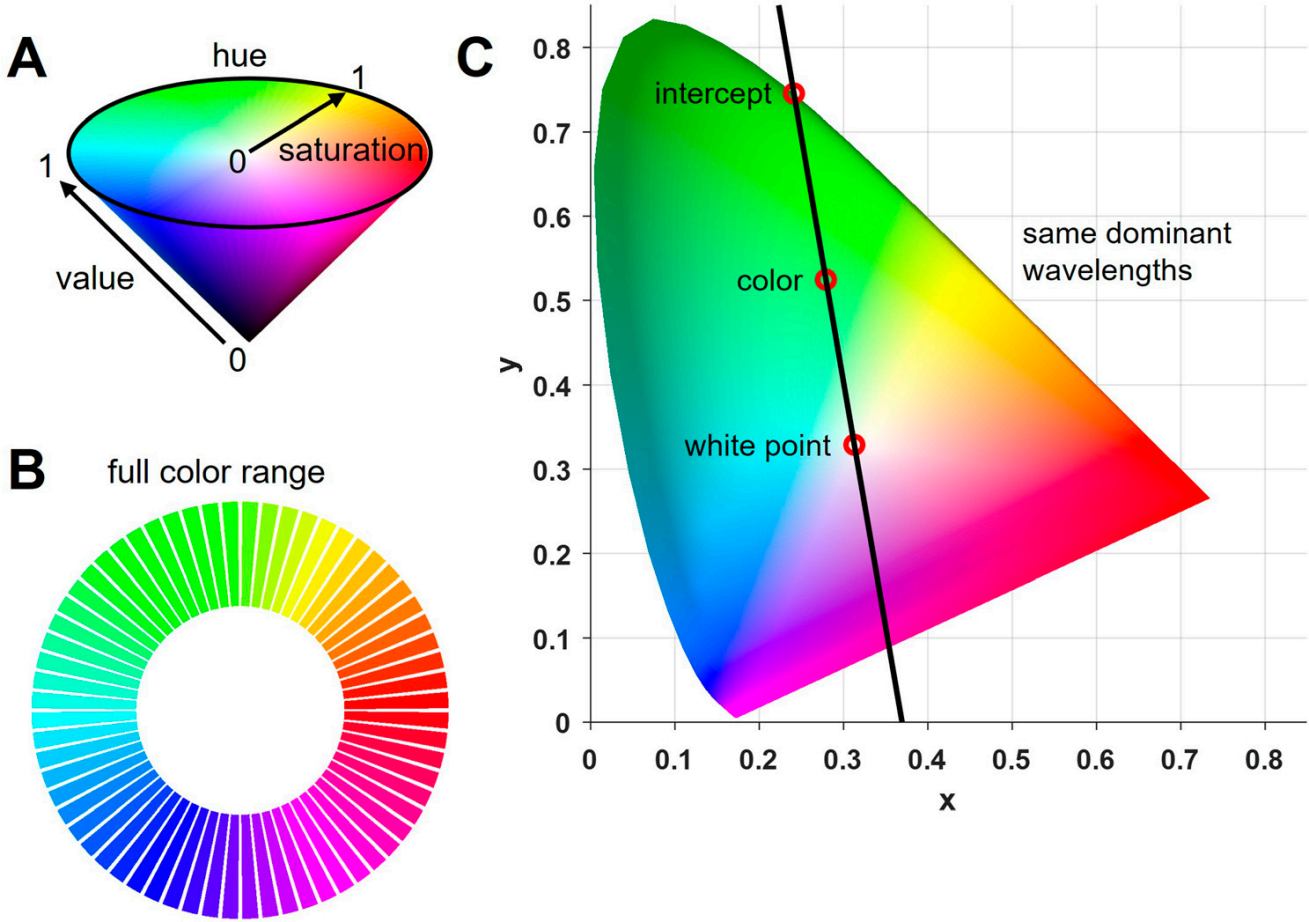
Experimental colors. **(A)** A full range of colors, or gamut, was obtained by diving hue in the HSV color space into 64 equidistant angles and setting saturation and value to 1, thereby creating full spectral colors. **(B)** Full color range (gamut) used in the experiment (64 colors). **(C)** CIE chromaticity diagram including the white point D65 and an example color (derived from converting the RGB values into CIE-XYZ values) from the full gamut. A linear function was fitted through both to create an intercept with the outer spectral color line. All colors on this line have the same dominant wavelength which can be obtained by identifying the intercept and its corresponding wavelength in standardized tables.

The experimental colors used in this study were generated using MATLAB, where they were defined in the HSV color space. Saturation and value were set to 1, because only clear spectral colors were needed (Figure 1A). Hue was split into 64 equally spaced colors using the full range to create a gamut (full circular color range, Figure 1B). HSV values were then transformed into RGB values (for display purposes) using built-in MATLAB functions, and further into the CIE-XYZ values to calculate the theoretical dominant wavelength of each color (CIE, 1932). CIE, short for ‘international commission of illumination’, represents efforts made to develop a color space that is standardized to human perception. By matching a given light source with three independent red, green and blue lights, color matching functions were obtained that represent the standard observer’s perceived proportions of each light in any given color. By doing so, a color space that matches the physical properties of a color to the human psychophysical perception was created (Shaw and Fairchild, 2002). For the present study, calculations of dominant wavelengths were based on the D65 white point (for stimuli displayed on a computer monitor) and x and y coordinates of each experimental color (derived from CIE-XYZ values). By fitting a linear function to all experimental colors (going through both, the respective color and D65), the dominant wavelength could be determined from the intercept of the resulting line and the spectral color line (Figure 1C; see Malacara, 2002). The normative data about which wavelength corresponds to which exact point on the spectral color line was obtained from standardized tables (Stockman and Sharpe, 2000).

**FIGURE 2:**
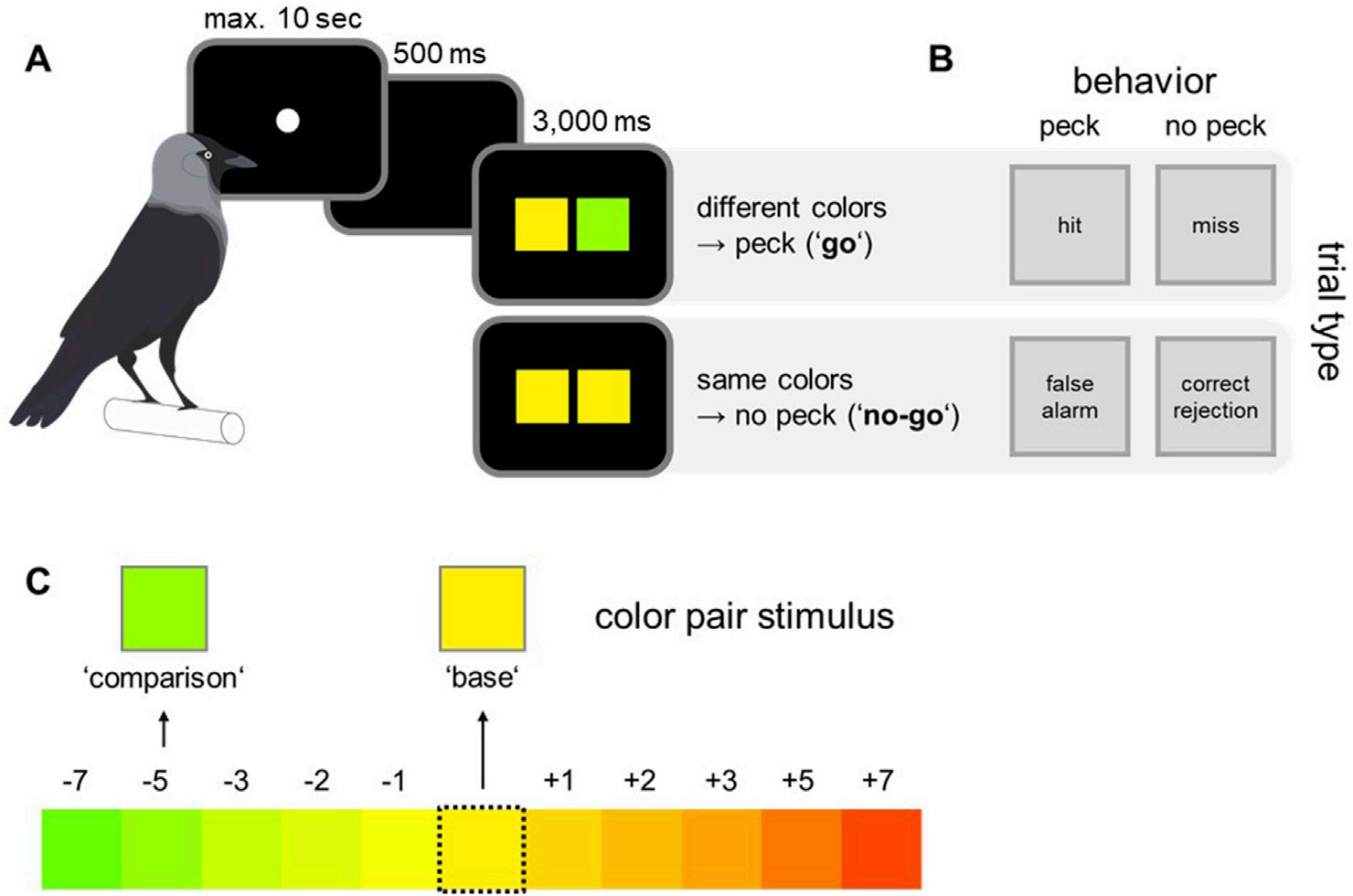
Experimental paradigm, overview of behavioral responses and color pair selection. **(A)** The birds were trained on a go/no-go paradigm. To start a trial, the birds pecked the initialization stimulus. In “go” trials the correct behavior was to directly peck on the monitor, whereas in “no-go” trials the birds had to wait for a second test array before responding. **(B)** Depending on experimental condition and behavioral response, four distinct response types could be differentiated: hit, miss, false alarm, and correct rejections. **(C)** Out of the whole gamut, one color was randomly selected as base color. The corresponding comparison color was randomly selected within a range of 7 adjacent colors in both directions of the color wheel, resulting in 10 distinct color steps, representing distinct color pair differences.

To compare the values of calculated dominant wavelengths with the actual colors displayed on the monitor, post-hoc measurements were conducted using a spectroradiometer (Konica Minolta Chroma Meter CS-150). For these measurements, color stimuli were presented on the monitor in the same way as they were presented as sample stimulus in the behavioral paradigm (the same color shown twice as in the ‘no go’ condition, Figure 2A). For each color, 10 measurements of wavelength, luminance and saturation were taken in darkness, and averaged.

### Behavioral paradigm

The birds were trained on a go/no-go paradigm with specific color pairs. The task was to indicate whether two simultaneously presented colors were perceived as same or different. To obtain a food reward, the birds had to respond to color arrays showing different color hues or withhold from pecking until a second test array appeared if the two presented colors were different (Figure 2A).

To initiate each trial, the birds had to peck on a small white circle (initialization stimulus, Figure 2A). After a delay of 500 ms, two colored squares were displayed for 3,000 ms (size: five by 5 cm, left: base color; right: comparison color). In such a go/no go paradigm, four response types could be distinguished (Figure 2B). If both squares were of different color (go trials), the correct response was to peck anywhere on the monitor (hit). In no-go trials, the birds had to wait for a second test array to be presented (correct rejection) before pecking (color squares of test 2 were always different). The birds were rewarded for correct pecks and received a brief monitor flash and short time-out as error signal if they pecked in no-go trials (false alarm) or missed to respond in go trials (miss). Since the second test array in no-go trials always contained different colors, the birds could receive a reward in these trials as well, which ensured that the birds did not simply stop responding in no-go trials.

The number of go and no-go trials was balanced in each experimental session and the order of trial types was pseudo-randomized. Further, the color pairs for each trial were chosen pseudo-randomly. Pseudo-randomization was done to ensure that the same trial type or color combination did not occur more than three times consecutively. Every color pair consisted of a base color, which was chosen out of the whole range of all 64 colors, and a comparison color (Figure 2C). The comparison color was chosen out of a defined set of 10 colors for every base color (resulting in 10 distinct color steps, representing distinct color pair differences). This set was defined by the relative distance of all comparison colors to the respective base color. Each comparison color was either 1, 2, 3, 5 or 7 steps away from the base color in both directions of the color wheel (Figure 2C).

### Data collection and analysis

A period of training was done in the beginning to ensure that the birds perform the go/no go task. They were only presented with three colors that were chosen out of the full range of 64 colors with a maximum distance in hue. The training period ended, once the birds performed on this above 60% correct responses (overall, hits and correct rejections). For the final collection of data, the whole gamut of colors was used as base color from the beginning. The comparison color steps were introduced sequentially; first the comparison colors being ±7 steps away (bidirectional), then those ±5 steps from the base color and finally the color steps ±1, ±2, and ±3 steps were introduced together. While new color steps were introduced, the former color steps were still probed in a randomized fashion to keep the animals engaged in the task.

All analyses were performed with MATLAB, using custom code and the Curve Fitting Toolbox. If not indicated otherwise, performance was calculated as mean percent correct responses, with chance level being at 50%. Receiver operating characteristic curves (ROC) were modeled to quantify the deviation of the birds’ performance from chance by calculating the area under the curve. ROC curves were based on logistic regression of the data. Cochran’s Q-Test was used to test for differences in distribution of correct responses per color. Discrimination curve modelling was done by fitting a non-linear regression to the performance data using the Curve Fitting Toolbox. Post hoc power analysis was done using G*Power 3.1. To quantify the contribution of each of the measured physical parameters (wavelength, luminance, saturation) to the discrimination performance of the birds, coefficients of multiple correlation and the corresponding *R* ^2^ values were calculated. In other words, the performance of the birds was correlated with each of the color stimulus parameters, corrected for the intercorrelations between those parameters.

## Results

### Calculated and measured dominant wavelengths deviated similarly from a linear color distribution

**FIGURE 3:**
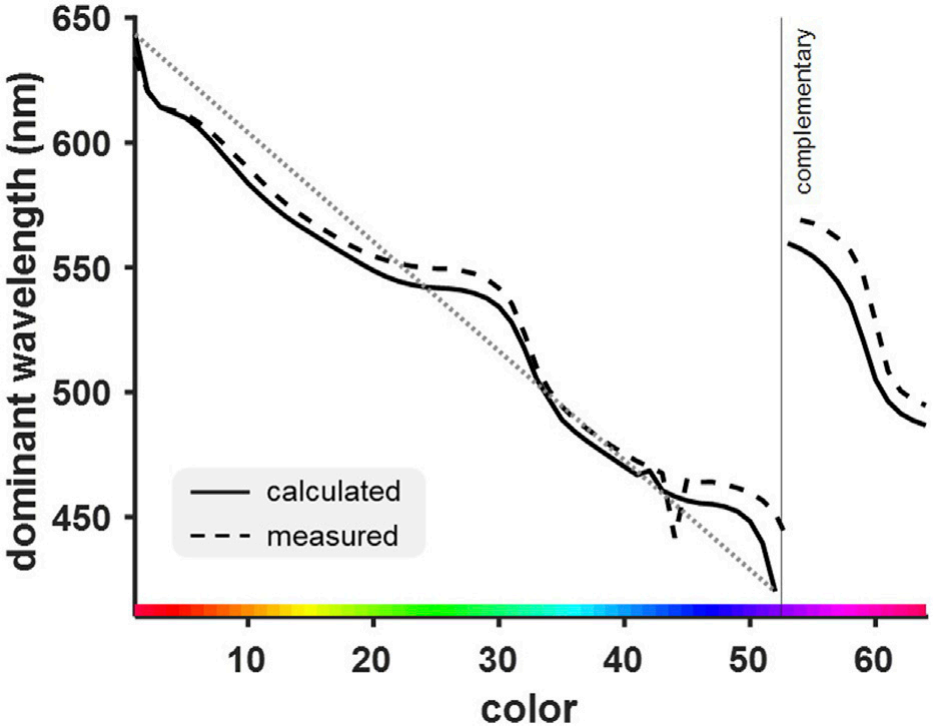
Calculated and measured dominant (and complementary) wavelengths of the experimental colors deviated from an ideal linear relationship (grey dotted line) between wavelength and color. This deviation was more pronounced for measured wavelengths, which further included the color distortion of the computer monitor. Reduced slopes for certain green and blue hues indicate a higher similarity of colors within these wavelength ranges (color ID 22-34, and 41-52, respectively).

The dominant wavelength of each color used in this study was calculated and compared to the wavelength obtained from measurements in the experimental setup (see Supplementary Table S2). This enabled a comparison between calculated and actual dominant wavelength to assess in which ways the experimental monitor distorted the originally intended presentation of the colors. A dominant wavelength could be calculated for most experimental colors (solid curve, Figure 3) except some purple hues for which only complementary instead of dominant wavelengths could be calculated (indicated via the vertical line in Figure 3; see Burger and Burge, 2015). The solid black curve in Figure 3 clearly shows that dominant wavelengths of almost all colors deviated from the ideal linear wavelength distribution shown by the dotted grey line. The dashed black curve represents the measured dominant wavelengths, which generally followed the same pattern but did not overlap with the calculated values. Thus, the color rendering of the monitor was not precise enough to produce the intended color according its RGB values. The largest deviation between both the measured and calculated wavelengths and the linear course was obvious for some green and blue hues (Figure 3 color IDs 22-34 and 41-52; see Supplementary Table S2). The reduced slope indicates that these colors were much more similar to one another than other colors, even though they were created aiming for identical wavelength differences.

### Birds showed color-dependent discrimination performance

**FIGURE 4:**
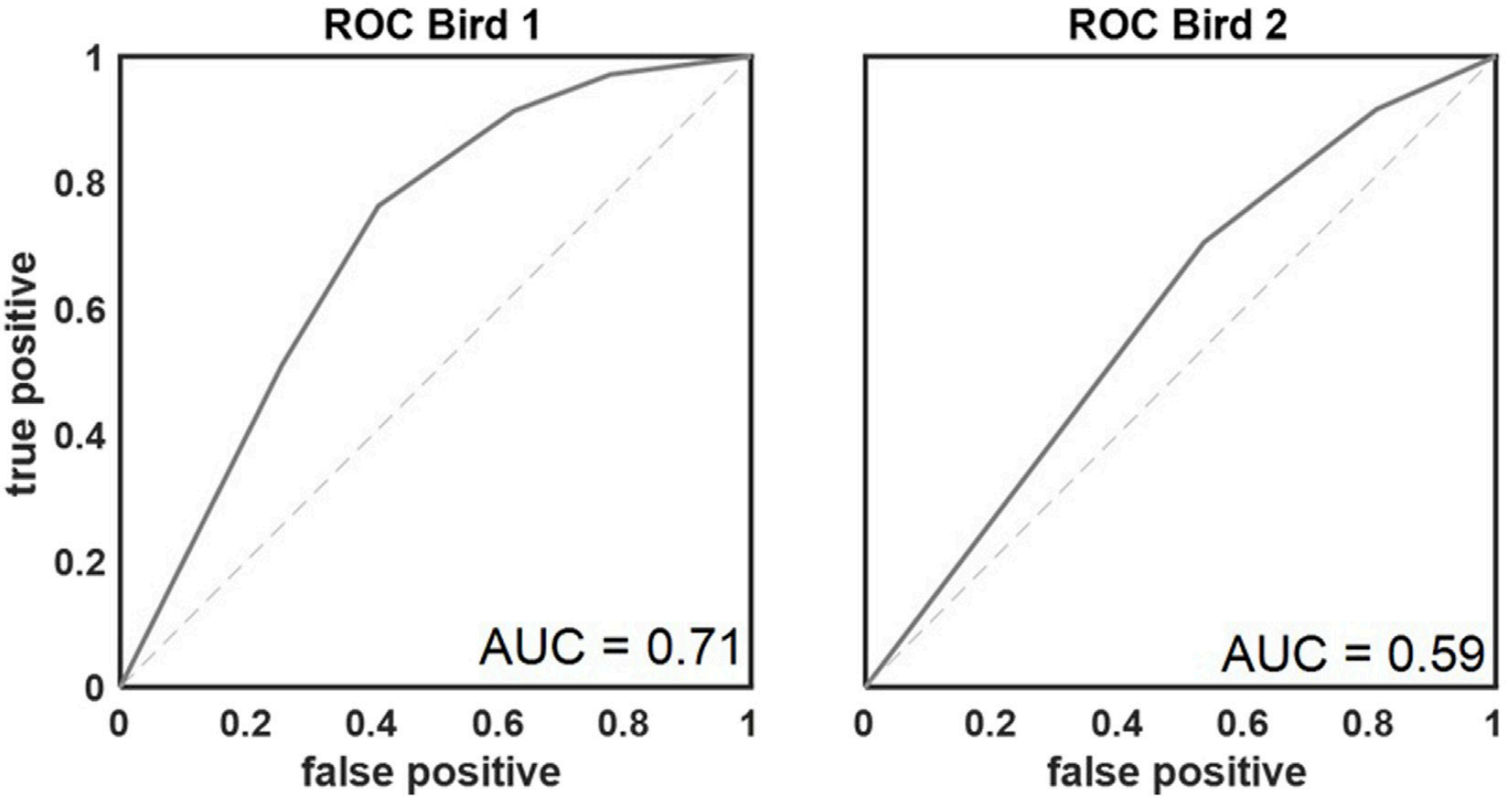
Receiver operating characteristic curves for both birds. AUROC values of 0.71 and 0.59 respectively demonstrated that the discriminatory performance of the birds was above chance (AUC = 0.5; indicated with dashed line).

In total, 82 sessions were analyzed in which the birds completed 24,426 color comparisons (bird 1, n = 20,365; bird 2, n = 4,061). The number of completed trials was similar across all base colors (Q (63) = 20.994, p = 1.00; average number of presentations per color ±standard deviation = 381.66 ± 11.28). Overall, both birds performed well above the chance level of 50% correct (Figure 4). As explained above, four different response types could be differentiated (Figure 2B). Performance of the birds referred to the percentage of correct responses (hits and correct rejections; mean ± standard deviation in %: hit_bird1_ = 72.47 ± 10.68, hit_bird2_ = 76.11 ± 8.62, correct rejection_bird1_ = 81.32 ± 8.46, correct rejection_bird2_ = 72.99 ± 5.64), which was higher than the percentage of incorrect responses (misses and false alarm; mean ± standard deviation in %: miss_bird1_ = 27.52 ± 10.68, miss_bird2_ = 5.8 ± 11.11, false alarm_bird1_ = 18.68 ± 8.46, false alarm_bird2_ = 6.5 ± 11.84). In total, correct responses (hit and correct rejection) were most frequent (76.77% of all responses). The proportion of correct responses was further quantified by calculating the area under the receiver operating characteristics curves (Figure 4) resulting in values of 0.71 (bird 1) and 0.59 (bird 2).

### Behavioral performance was color-dependent and decreased with color pair difference

**FIGURE 5:**
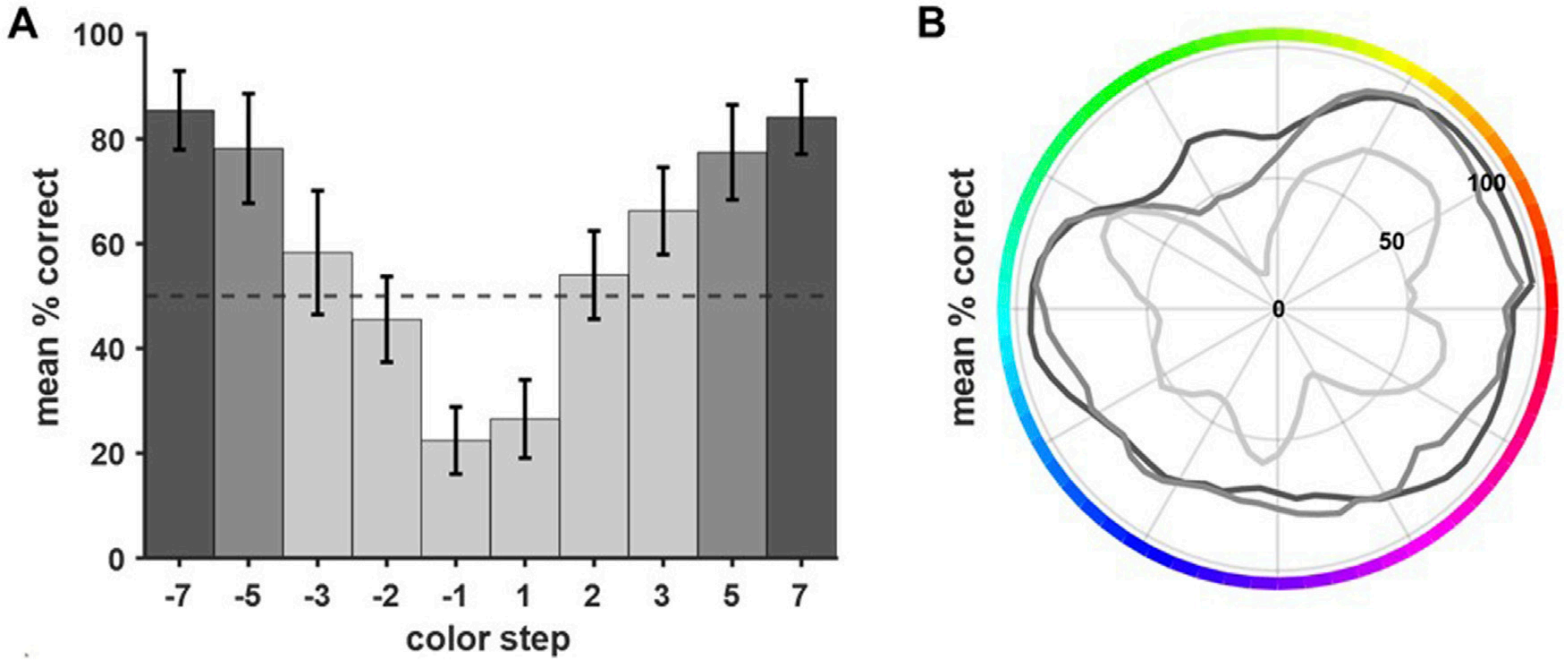
Performance differed depending on the distance between base and comparison color and the base color itself. **(A)** The average performance was calculated per color step (pooled across all colors, error bars show SEM). Performance decreased the closer base color and comparison color were (irrespective of the direction of shift). For color comparisons closer than three steps, the performance dropped below chance level. **(B)** In general, the performance per base color decreased with decreasing difference between base and comparison color (grey shading indicates color steps, dark grey = ±7, grey = ±5, and light grey = ±3, ±2, ±1). Clear differences in performance per base color became apparent for the more difficult comparisons (i.e., color steps ±3, ±2, ±1). Here, the birds showed clear drops in performance for close comparisons within the green, the blue, and the purple range.

As explained above, each base color was combined with 10 different comparison colors. Due to the training history (color steps were introduced successively starting with the largest color steps of ±7) the number of trials per color step differed. The final data set included all trials with the whole gamut used as base colors (see Supplementary Table S1). The average performance decreased as the distance between base and comparison color became smaller (Figure 5A). For comparison colors closer than 3 steps, the performance dropped below chance level (light grey, Figure 5). The symmetrical distribution of performance levels argued against different levels of difficulty due to clockwise or counterclockwise comparison color shifts. Behavioral performance differed not only per color step but also as a function of specific base colors. Figure 5B shows the performance per base color pooled across specific comparison color steps (light grey: ±1/2/3; grey: ±5; dark grey: ±7). Overall, performance differed significantly between the different target colors (all data pooled, Q (63) = 834.4025, p < 0.001). The outer, dark grey line contains all trials with color steps of ±7. The performance was quite similar for different base colors, showing only slightly reduced performance in the green and blue range. The middle grey line visualizes the performance in trials with intermediate color steps (±5). Overall performance was reduced but again showed only minor differences between specific base colors. Clear color-dependent differences became apparent for the closest color comparisons (color steps ±3, ±2 and ±1, inner light grey line). Clear drops in performance could be seen for green, blue, and purple hues.

### Discrimination curve modelling revealed discrimination abilities depended on base color

**FIGURE 6:**
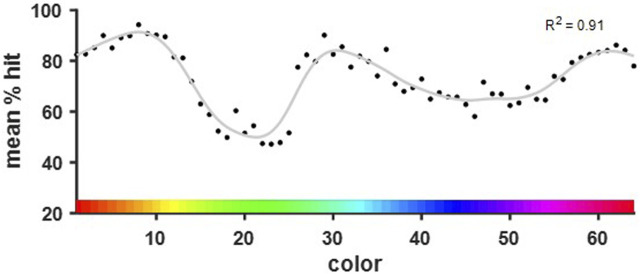
Discrimination curve fitted to the performance per base color over all color steps. The three peaks of the curve were found in the orange, cyan, and purple spectra. The local minima corresponded with the green and blue hues that were shown to differ the least in their dominant wavelengths.

A discrimination curve was modeled to fit the behavioral data of all completed color comparisons. A post-hoc analysis revealed that a discrimination curve fitted to the present data set can reveal differences in color discrimination performance with a power of (1-β) = 0.93. A three peaked curve was found to fit the data best (*R* ^2^ = 0.91, RMSE = 4.25, df = 49.57). The peaks were found in the orange, cyan, and purple spectra (Figure 6). Lowest performance was found for green and blue hues. These colors correspond to those found to be much more similar to one another based on their dominant wavelengths (see Figure 3).

### Dominant wavelength represented the physical parameter most predictive of the behavioral performance

**FIGURE 7:**
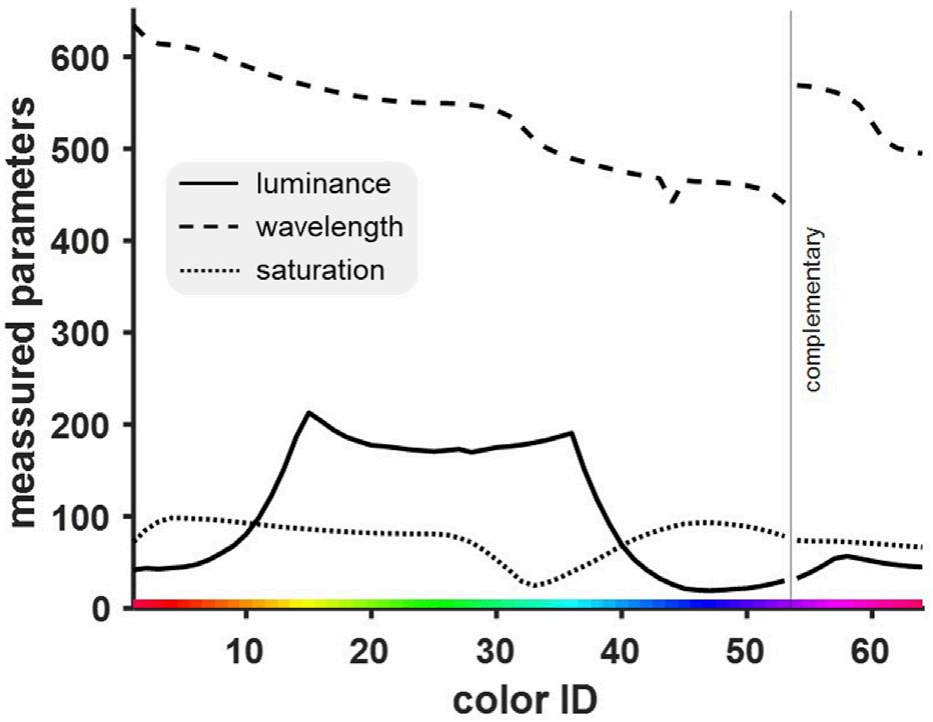
Distribution of three different physical measures of color [luminance (cd/m^2^), wavelength (nm), saturation (%)]. The three measured parameters differed in their course and from the intended linear color display. We expected to find an equidistant distribution of wavelengths (see Figure 3) and both saturation and luminance to be constant at their respective maximum value. While saturation was relatively constant, luminance was relatively high for one-half of the colors and relatively low for the others.

Finally, to confirm that the obtained discrimination curve was indeed a function of the birds’ abilities to distinguish the presented wavelengths rather than their luminance or saturation, correlation and analysis of shared variance were conducted. Figure 7 shows the distribution of all three measured characteristics of presented colors (luminance, wavelength and saturation) across the entire stimulus set.

Standard correlation analysis showed a high positive correlation between performance and dominant wavelength (r = 0.402, p = 0.001). The other two variables failed to reach significance in their correlation with performance and were comparatively small, with saturation (r = −0.064, p = 0.614) being slightly more relevant than luminance (r = −0.044, p = 0.729). This showed that with 16% shared variance (R^2^_wavelength_ = 0.16), the wavelengths of the presented colors had the biggest influence on discrimination performance (R^2^_saturation_ = 0.004, R^2^_luminance_ = 0.002).

Since the physical measures of color represent integral stimulus properties that are related with each other, a partial correlation was calculated in addition. This changed the absolute values of the correlations, but not the pattern. With a shared variance of now 26%, wavelength remained the most influential (r = 0.512, p < 0.0001, *R* ^2^ = 0.26). The negative correlations of saturation (r = −0.331, p = 0.009, *R* ^2^ = 0.11) and luminance (r = −0.306, p = 0.01, *R* ^2^ = 0.09) were bigger as well and reached significance, but these variables were still not as influential as the wavelengths of the colors.

## Discussion

Our study aimed to find a psychometric color discrimination curve for jackdaws to better interpret behavioral results obtained from various experimental paradigms (using color stimuli). In addition, we performed detailed measurements of rendered colors to evaluate color accuracy of stimulus generation and computer monitor display (i.e., dominant wavelength, luminance, saturation). We found that the rendered colors deviated from the intended linear color range, resulting in color ranges comprising more similar or more distinct color hues. The birds were able to perform above chance level and, as expected, performance dropped when hues became more similar to each other. Overall, birds mostly relied on hue to differentiate color pairs. Drops in discrimination performance mostly correlated with physical stimulus properties, which again highlights the importance to also include detailed stimulus information when interpreting behavioral findings (also see Bae et al., 2014).

Overall, the birds performed well at the task and were able to discriminate the colors above chance level. For distant comparison colors (color steps ±5, ±7), performance was very similar across all colors, with about 80 percent correct responses. When looking at the closest color steps (±1, ±2, ±3), performance dropped below chance level. A closer look at the performance revealed a color dependency for the discrimination ability of adjacent color pairs (Figure 5B). Here, behavioral performance correlated strongly with the physical stimulus properties, namely, dominant wavelength. Thus, irregularities of the hue distribution had a strong effect on discrimination ability which became more pronounced for close and especially adjacent color pairs. In general, birds mostly relied on wavelength to discriminate color pairs and were less affected by the other physical stimulus properties, i.e., luminance and brightness. This supports earlier findings, such as in budgerigars, where it has been shown that the birds are able to discriminate colors regardless of their brightness level (Goldsmith and Butler, 2005). For saturation previous findings indicate that chicks generalize across hues and saturations in the same way (Scholtyssek et al., 2016). Thus, those seem to be related qualities of color that can independently but similarly contribute to bird color discrimination. In our study, the birds seemed to have identified hue as the defining characteristic of the color and thus used this chromatic dimension to base their responses on rather than saturation. Of course, as ‘integral stimulus parameters’ (as described in CIE, 1932), hue, saturation and brightness are highly correlated and cannot easily be investigated individually (Burns and Shepp, 1988; Xie and Fairchild, 2023). Interestingly, while wavelength and discrimination performance had a positive correlation, the correlation of both luminance and saturation with performance was negative, which shows, that the pattern of performance did not coincide with the pattern of either of those parameters. This indicates even further that mostly the wavelength of the colors was informative to the birds.

We fitted a discrimination curve to the data, which revealed three peaks of increased discrimination ability in jackdaws. Although this would generally align with peaks of receptor sensitivity in the visual system as shown in previous work (Hart and Hunt, 2007), our non-linear stimulus distribution complicates this interpretation. We found that the minima of the discrimination curve map onto those wavelengths with a reduced distance (between them). Thus, this can most likely be explained by a higher physical similarity of the color pairs tested instead of reduced sensitivity in the specific color range. This interpretation was supported by findings from a small side study with humans, in which a small sample of human participants was tested in the same experimental setup under the exact same conditions and the pattern of performance we found was very similar to the birds. A detailed description of this preliminary study goes beyond the scope of this paper; however, it can be mentioned that the human subjects showed a very similar discrimination curve despite profound differences in both visual systems (see Supplementary Figure S1). Both birds and humans were thus equally affected in their color discrimination by the non-linear distribution of wavelengths.

Normally, differences in color perception between human and avian subjects must be expected given the differences in the visual systems of birds and humans (tetrachromatic vs trichromatic vision, Kelber, 2019; Kelber et al., 2003). However, in terms of perceptual ability, humans and birds seem to have evolved similarly. Both birds and humans are highly visual animals (Hutmacher, 2019; Jones et al., 2007; Martin, 2012). Being able to discriminate a wide range of colors allowed them to be successful in their respective environments. For instance, heightened color discrimination through tri- and tetrachromacy was relevant in finding the ripest food containing the most calories (Smith et al., 2003; Stoddard et al., 2020). This put similar pressure on birds and early humans, with which a similar color discrimination ability evolved, even though their brains are vastly different (Lefebvre et al., 2004; Osorio and Vorobyev, 2008). Not only in color perception, but also in many cognitive tasks some bird families, such as corvids, show similar abilities to primates and monkeys, despite their different brains. We can see, for example, similarities in cognitive control (Balakhonov and Rose, 2017) and working memory capacity limitations (Hahn et al., 2021).

Still, even though birds and humans are similar in color perception abilities, the underlying physiology differs. One major difference is the presence of a fourth receptor type in the avian retina, sensitive to ultraviolet wavelengths (Kelber, 2019), which adds another dimension to the bird color space (Stoddard et al., 2020) and might even influence perception of other colors (Emmerton and Delius, 1980). Consequently, to examine pure perceptual color discrimination, adapted experimental hardware (e.g., monitors capable of rendering UV with precise color display) and a better knowledge of the specific avian photoreceptor pigment sensitivity (e.g., as for pigeons in Bowmaker et al., 1997, hummingbirds in Huth and Burkhardt, 1972 or Bennet et al., 1996 in zebra finches) would be necessary. A previous study on hummingbird color vision established an innovative tetrachromatic color space, adapted to hummingbird cone sensitivities and their ability to discriminate non-spectral colors containing a UV component (Stoddard et al., 2020). An approach like this allows to fully map the color discrimination ability of an avian species and should be obtained for other bird species as well. However, up to now color cone sensitivities are only known for few bird species such as pigeons (Bowmaker et al., 1997), chickens (Osorio et al., 1999), and hummingbirds (Herrera et al., 2008) and in most experimental setups the UV component of color stimuli cannot easily be incorporated. An advantage of the hummingbird study by Stoddard et al. (2020) was their use of innovative light sources displaying the color stimuli. In their ‘TetraColorTubes’ individual LEDs of red, green, blue and UV could be illuminated to create a unique color comprised of different proportions of those colors, adapted to the specific spectral sensitivities of the hummingbirds (Stoddard et al., 2020). So far, to our knowledge, no commercially available PC screen can display UV components. Further developing such systems to then gain better understanding of other bird species’, including jackdaws, color discrimination abilities, would be an interesting avenue for the future.

To examine our color stimuli, we used the CIE color space, even though it originally was made for humans. We found that it is beneficial for a characterization of colors because it allows to connect a RGB color with its underlying physical wavelength. CIE-XYZ, as a device independent standard observer model, allows to draw conclusions about colors regardless of their medium of presentation. From the three-dimensional CIE-XYZ space, the two-dimensional chromaticity of all colors was calculated as a luminance-independent measure (Shaw and Fairchild, 2002). Further transformation of the data allowed us to calculate the corresponding dominant wavelength for each experimental color (Malacara, 2002). This approach revealed an already non-uniform distribution of wavelengths despite the uniform hue distribution within the HSV color space for our intended colors. Thus, the strongest deviation from the ideal linear color range was due to the transformation of HSV colors into their corresponding RGB values instead of imprecise color rendering. Calculated and measured wavelengths coincided largely, which again emphasizes the need for better controlled color generation procedures and additional post-hoc measurements of experimental colors.

Our findings are crucial for future studies on color perception and highlight once again that precise stimulus control and even the examination of physical stimulus parameter are necessary (Bae et al., 2014; Hardman et al., 2017). However, as mentioned in the introduction, more detailed knowledge on color discrimination abilities is also of relevance for work related to cognition. For example, change detection paradigms need to consider that certain color pairs might be easier to differentiate. Consequently, the specific color pair could affect the number of items for which color changes can be correctly identified. Similarly, a recent study in jackdaws revealed that certain colors seem to be memorized more precisely and more easily than others (Apostel et al., 2023b) – a finding also known for primates and humans (Bae et al., 2015; Panichello et al., 2019) and potentially of consequence for future studies. Although this seems to represent a behavioral strategy to balance memory precision and capacity limitations, the precise position of attractor colors could be related to color rendering details and or cone sensitivity peaks (Apostel et al., 2023b).

In conclusion, using colorful stimuli in a neuroscience experiment allows for a rich and naturalistic stimulus set. Jackdaws can readily discriminate different colors based on their hue. Yet, one needs to examine physical stimulus parameter and to consider the specific distribution of wavelengths when interpreting the results of a study involving colorful stimuli.

## Data Availability

The original contributions presented in the study are included in the article/Supplementary Material, further inquiries can be directed to the corresponding authors.
