# Supplementary material for: “Distribution of dominant wavelengths predicts jackdaw (Corvus monedula) color discrimination performance”
Source: Front Physiol. 2025 Feb 20;16:1543469. doi: 10.3389/fphys.2025.1543469 (PMC11882508; doi:10.3389/fphys.2025.1543469)
Supplement: Supplementary file 1 [file Table1.docx]

**Appendix**

Table S1: Number of trials, mean performance and standard deviation per color and color step. Each trial consisted of a pseudo-randomized base color (col ID) and a comparison color that was either 7, 5, 3, 2 or 1 color steps away in both directions of the color wheel. Due to those being added to the task sequentially, color steps 7 and 5 have a higher number of trials than 3, 2, and 1.

| **col  ID** | **number of trials** | | | | | **mean performance (% correct)** | | | | | **SEM performance** | | | | |
| --- | --- | --- | --- | --- | --- | --- | --- | --- | --- | --- | --- | --- | --- | --- | --- |
|  | **±7** | **±5** | **±3** | **±2** | **±1** | **±7** | **±5** | **±3** | **±2** | **±1** | **±7** | **±5** | **±3** | **±2** | **±1** |
| **1** | 166 | 94 | 66 | 29 | 31 | 90.96 | 87.23 | 69.70 | 55.17 | 70.97 | 2.23 | 3.46 | 5.70 | 9.40 | 8.29 |
| **2** | 178 | 84 | 70 | 30 | 25 | 93.82 | 88.10 | 74.29 | 66.67 | 40.00 | 1.81 | 3.55 | 5.26 | 8.75 | 10 |
| **3** | 176 | 87 | 65 | 27 | 24 | 89.77 | 85.06 | 73.85 | 74.07 | 62.5 | 2.29 | 3.84 | 5.49 | 8.59 | 10.09 |
| **4** | 187 | 73 | 55 | 31 | 29 | 93.58 | 84.93 | 89.09 | 77.42 | 62.07 | 1.80 | 4.22 | 4.24 | 7.63 | 9.17 |
| **5** | 180 | 98 | 63 | 25 | 27 | 91.67 | 89.8 | 82.54 | 80.00 | 59.26 | 2.07 | 3.07 | 4.82 | 8.16 | 9.64 |
| **6** | 184 | 87 | 56 | 24 | 25 | 91.85 | 94.25 | 91.07 | 87.50 | 60.00 | 2.02 | 2.51 | 3.85 | 6.90 | 10.00 |
| **7** | 186 | 91 | 58 | 29 | 37 | 94.62 | 92.31 | 89.66 | 82.76 | 54.05 | 1.66 | 2.81 | 4.03 | 7.14 | 8.31 |
| **8** | 188 | 82 | 61 | 30 | 30 | 95.74 | 96.34 | 91.80 | 83.33 | 73.33 | 1.48 | 2.09 | 3.54 | 6.92 | 8.21 |
| **9** | 185 | 88 | 60 | 21 | 28 | 92.97 | 88.64 | 93.33 | 80.95 | 67.86 | 1.88 | 3.40 | 3.25 | 8.78 | 8.99 |
| **10** | 189 | 86 | 65 | 29 | 28 | 94.18 | 93.02 | 84.62 | 75.86 | 53.57 | 1.71 | 2.76 | 4.51 | 8.09 | 9.60 |
| **11** | 184 | 86 | 58 | 22 | 26 | 91.30 | 81.40 | 82.76 | 81.82 | 65.38 | 2.08 | 4.22 | 5.00 | 8.42 | 9.51 |
| **12** | 171 | 69 | 62 | 25 | 27 | 79.53 | 86.96 | 79.03 | 68.00 | 29.63 | 3.09 | 4.08 | 5.21 | 9.52 | 8.96 |
| **13** | 176 | 98 | 64 | 24 | 25 | 73.86 | 75.51 | 68.75 | 66.67 | 52.00 | 3.32 | 4.37 | 5.84 | 9.83 | 10.20 |
| **14** | 165 | 88 | 61 | 25 | 31 | 75.15 | 73.86 | 62.30 | 48.00 | 67.74 | 3.37 | 4.71 | 6.26 | 10.20 | 8.53 |
| **15** | 181 | 80 | 67 | 28 | 28 | 69.06 | 70.00 | 64.18 | 53.57 | 50.00 | 3.45 | 5.16 | 5.90 | 9.60 | 9.62 |
| **16** | 178 | 87 | 61 | 24 | 24 | 71.91 | 71.26 | 59.02 | 79.17 | 62.50 | 3.38 | 4.88 | 6.35 | 8.47 | 10.09 |
| **17** | 199 | 84 | 65 | 25 | 29 | 66.33 | 60.71 | 50.77 | 56.00 | 68.97 | 3.36 | 5.36 | 6.25 | 10.13 | 8.74 |
| **18** | 181 | 84 | 61 | 27 | 24 | 74.59 | 67.86 | 59.02 | 51.85 | 54.17 | 3.25 | 5.13 | 6.35 | 9.80 | 10.39 |
| **19** | 173 | 79 | 61 | 28 | 26 | 75.72 | 67.09 | 54.10 | 46.43 | 57.69 | 3.27 | 5.32 | 6.43 | 9.60 | 9.88 |
| **20** | 184 | 92 | 60 | 25 | 24 | 80.98 | 64.13 | 46.67 | 44.00 | 45.83 | 2.90 | 5.03 | 6.49 | 10.13 | 10.39 |
| **21** | 174 | 78 | 72 | 27 | 25 | 78.74 | 64.10 | 44.44 | 44.44 | 48.00 | 3.11 | 5.47 | 5.90 | 9.75 | 10.20 |
| **22** | 183 | 80 | 61 | 26 | 28 | 70.49 | 60.00 | 57.38 | 57.69 | 57.14 | 3.38 | 5.51 | 6.38 | 9.88 | 9.52 |
| **23** | 175 | 81 | 63 | 32 | 28 | 70.29 | 69.14 | 63.49 | 46.88 | 32.14 | 3.46 | 5.16 | 6.11 | 8.96 | 8.99 |
| **24** | 184 | 93 | 66 | 23 | 27 | 69.02 | 65.59 | 66.67 | 60.87 | 55.56 | 3.42 | 4.95 | 5.85 | 10.41 | 9.75 |
| **25** | 181 | 88 | 61 | 27 | 24 | 65.19 | 48.86 | 59.02 | 59.26 | 45.83 | 3.55 | 5.36 | 6.35 | 9.64 | 10.39 |
| **26** | 179 | 81 | 64 | 28 | 28 | 67.60 | 67.90 | 71.88 | 75.00 | 46.43 | 3.51 | 5.22 | 5.66 | 8.33 | 9.60 |
| **27** | 183 | 96 | 54 | 26 | 28 | 68.85 | 71.87 | 61.11 | 61.54 | 64.29 | 3.43 | 4.61 | 6.70 | 9.73 | 9.22 |
| **28** | 176 | 88 | 52 | 30 | 30 | 80.68 | 70.45 | 69.23 | 56.67 | 50.00 | 2.98 | 4.89 | 6.46 | 9.20 | 9.28 |
| **29** | 185 | 87 | 63 | 27 | 25 | 86.49 | 88.51 | 85.71 | 70.37 | 60.00 | 2.52 | 3.44 | 4.44 | 8.96 | 10.00 |
| **30** | 171 | 94 | 57 | 23 | 27 | 90.06 | 82.98 | 75.44 | 69.57 | 59.26 | 2.29 | 3.90 | 5.75 | 9.81 | 9.64 |
| **31** | 178 | 87 | 60 | 28 | 28 | 89.33 | 81.61 | 76.67 | 75.00 | 35.71 | 2.32 | 4.18 | 5.51 | 8.33 | 9.22 |
| **32** | 173 | 85 | 68 | 26 | 25 | 91.91 | 85.88 | 67.65 | 57.69 | 56.00 | 2.08 | 3.80 | 5.72 | 9.88 | 10.13 |
| **33** | 179 | 81 | 61 | 25 | 25 | 83.80 | 88.89 | 68.85 | 68.00 | 68.00 | 2.76 | 3.51 | 5.98 | 9.52 | 9.52 |
| **34** | 174 | 88 | 62 | 28 | 24 | 89.08 | 76.14 | 67.74 | 78.57 | 41.67 | 2.37 | 4.57 | 5.99 | 7.90 | 10.28 |
| **35** | 182 | 90 | 54 | 27 | 29 | 84.07 | 83.33 | 72.22 | 77.78 | 62.07 | 2.72 | 3.95 | 6.15 | 8.15 | 9.17 |
| **36** | 174 | 92 | 59 | 31 | 26 | 81.03 | 76.09 | 76.27 | 51.61 | 57.69 | 2.98 | 4.47 | 5.59 | 9.12 | 9.88 |
| **37** | 181 | 81 | 65 | 29 | 24 | 76.24 | 70.37 | 64.62 | 55.17 | 58.33 | 3.17 | 5.11 | 5.98 | 9.40 | 10.28 |
| **38** | 181 | 84 | 62 | 31 | 34 | 73.48 | 73.81 | 69.35 | 58.06 | 55.88 | 3.29 | 4.83 | 5.90 | 9.01 | 8.64 |
| **39** | 174 | 87 | 59 | 32 | 23 | 75.86 | 74.71 | 72.88 | 50.00 | 34.78 | 3.25 | 4.69 | 5.84 | 8.98 | 10.15 |
| **40** | 194 | 92 | 53 | 30 | 28 | 75.77 | 65.22 | 67.92 | 63.33 | 60.71 | 3.08 | 4.99 | 6.47 | 8.95 | 9.40 |
| **41** | 190 | 86 | 72 | 29 | 27 | 76.84 | 69.77 | 51.39 | 58.62 | 62.96 | 3.07 | 4.98 | 5.93 | 9.31 | 9.47 |
| **42** | 183 | 88 | 67 | 26 | 29 | 75.41 | 71.59 | 52.24 | 65.38 | 48.28 | 3.19 | 4.84 | 6.15 | 9.51 | 9.44 |
| **43** | 180 | 84 | 64 | 25 | 30 | 75.00 | 70.24 | 64.06 | 56.00 | 36.67 | 3.24 | 5.02 | 6.05 | 10.13 | 8.95 |
| **44** | 183 | 89 | 63 | 25 | 28 | 77.05 | 69.66 | 76.19 | 60.00 | 42.86 | 3.12 | 4.90 | 5.41 | 10.00 | 9.52 |
| **45** | 191 | 86 | 60 | 34 | 27 | 67.54 | 62.79 | 73.33 | 61.76 | 44.44 | 3.40 | 5.24 | 5.76 | 8.46 | 9.75 |
| **46** | 178 | 98 | 60 | 28 | 30 | 67.98 | 58.16 | 55.00 | 57.14 | 70.00 | 3.51 | 5.01 | 6.48 | 9.52 | 8.51 |
| **47** | 171 | 74 | 60 | 27 | 24 | 68.42 | 70.27 | 56.67 | 62.96 | 62.50 | 3.57 | 5.35 | 6.45 | 9.47 | 10.09 |
| **48** | 181 | 95 | 63 | 29 | 29 | 67.96 | 66.32 | 55.56 | 58.62 | 68.97 | 3.48 | 4.87 | 6.31 | 9.31 | 8.74 |
| **49** | 180 | 92 | 59 | 25 | 23 | 66.67 | 59.78 | 72.88 | 56.00 | 43.48 | 3.52 | 5.14 | 5.84 | 10.13 | 10.57 |
| **50** | 181 | 92 | 64 | 29 | 26 | 78.45 | 67.39 | 71.88 | 55.17 | 46.15 | 3.06 | 4.91 | 5.66 | 9.40 | 9.97 |
| **51** | 181 | 81 | 61 | 23 | 24 | 73.48 | 71.60 | 62.30 | 60.87 | 41.67 | 3.29 | 5.04 | 6.26 | 10.41 | 10.28 |
| **52** | 180 | 88 | 65 | 32 | 28 | 77.22 | 84.09 | 66.15 | 56.25 | 57.14 | 3.13 | 3.92 | 5.91 | 8.91 | 9.52 |
| **53** | 169 | 76 | 58 | 29 | 29 | 76.33 | 88.16 | 56.9 | 55.17 | 44.83 | 3.28 | 3.73 | 6.56 | 9.40 | 9.40 |
| **54** | 181 | 86 | 69 | 30 | 26 | 83.43 | 75.58 | 59.42 | 56.67 | 38.46 | 2.77 | 4.66 | 5.95 | 9.20 | 9.73 |
| **55** | 166 | 81 | 61 | 31 | 24 | 83.13 | 77.78 | 67.21 | 64.52 | 41.67 | 2.92 | 4.65 | 6.06 | 8.74 | 10.28 |
| **56** | 169 | 83 | 57 | 26 | 25 | 78.70 | 65.06 | 64.91 | 61.54 | 40.00 | 3.16 | 5.27 | 6.38 | 9.73 | 10.00 |
| **57** | 170 | 82 | 57 | 26 | 26 | 79.41 | 63.41 | 59.65 | 53.85 | 61.54 | 3.11 | 5.35 | 6.56 | 9.97 | 9.73 |
| **58** | 173 | 89 | 67 | 24 | 26 | 84.97 | 79.78 | 73.13 | 83.33 | 50.00 | 2.72 | 4.28 | 5.46 | 7.77 | 10.00 |
| **59** | 179 | 81 | 61 | 26 | 27 | 83.24 | 87.65 | 85.25 | 73.08 | 33.33 | 2.80 | 3.68 | 4.58 | 8.87 | 9.25 |
| **60** | 182 | 84 | 61 | 31 | 24 | 86.26 | 83.33 | 91.80 | 74.19 | 41.67 | 2.56 | 4.09 | 3.54 | 7.99 | 10.28 |
| **61** | 182 | 83 | 67 | 31 | 25 | 86.26 | 77.11 | 85.07 | 80.65 | 44.00 | 2.56 | 4.64 | 4.39 | 7.21 | 10.13 |
| **62** | 189 | 91 | 60 | 28 | 27 | 84.13 | 79.12 | 71.67 | 82.14 | 40.74 | 2.67 | 4.28 | 5.87 | 7.37 | 9.64 |
| **63** | 168 | 96 | 62 | 26 | 25 | 80.95 | 79.17 | 80.65 | 73.08 | 52.00 | 3.04 | 4.17 | 5.06 | 8.87 | 10.20 |
| **64** | 176 | 89 | 61 | 27 | 29 | 88.07 | 80.90 | 73.77 | 70.37 | 58.62 | 2.45 | 4.19 | 5.68 | 8.96 | 9.31 |

Table S2: Calculated and measured wavelengths (WL) and deviations from an ideal linear course (deviation linear) for both calculated and measured WL. Ideal linear course can only be properly conceptualized for dominant wavelengths (complementary wavelengths are indicated with ‘-‘). Color ID in reference to sorting of colors for Fig. 3. Note that deviation from ideal linear course is anchored to the minimum and maximum values of the calculated wavelengths.

| **col ID** | **calculated WL** | **deviation linear** | **measured WL** | **deviation linear** |
| --- | --- | --- | --- | --- |
| 1 | 643.4 | 0 | 634.37 | -9.03 |
| 2 | 620.7 | -18.3216 | 620.52 | -18.5858 |
| 3 | 614.3 | -20.3431 | 614.26 | -20.5515 |
| 4 | 612.1 | -18.1647 | 612.93 | -17.5873 |
| 5 | 610 | -15.8863 | 611.51 | -14.7131 |
| 6 | 606.2 | -15.3078 | 608.37 | -13.5588 |
| 7 | 601.1 | -16.0294 | 604.51 | -13.1246 |
| 8 | 595.2 | -17.551 | 599.76 | -13.5804 |
| 9 | 589.5 | -18.8725 | 594.76 | -14.2862 |
| 10 | 583.7 | -20.2941 | 589.94 | -14.8119 |
| 11 | 578.9 | -20.7157 | 584.81 | -15.6477 |
| 12 | 574.4 | -20.8373 | 579.9 | -16.2635 |
| 13 | 570.4 | -20.4588 | 575.56 | -16.3092 |
| 14 | 566.9 | -19.5804 | 571.81 | -15.765 |
| 15 | 563.8 | -18.302 | 568.4 | -14.8808 |
| 16 | 560.6 | -17.1235 | 565.23 | -13.7565 |
| 17 | 557.5 | -15.8451 | 562.18 | -12.5123 |
| 18 | 554.5 | -14.4667 | 559.18 | -11.2181 |
| 19 | 551.5 | -13.0882 | 556.7 | -9.40385 |
| 20 | 548.7 | -11.5098 | 554.53 | -7.27962 |
| 21 | 546.3 | -9.53137 | 552.9 | -4.61538 |
| 22 | 544.4 | -7.05294 | 551.58 | -1.64115 |
| 23 | 543.1 | -3.97451 | 550.54 | 1.613077 |
| 24 | 542.2 | -0.49608 | 549.93 | 5.297308 |
| 25 | 541.8 | 3.482353 | 549.52 | 9.181538 |
| 26 | 541.5 | 7.560784 | 549.43 | 13.38577 |
| 27 | 540.9 | 11.33922 | 548.72 | 16.97 |
| 28 | 539.7 | 14.51765 | 547.52 | 20.06423 |
| 29 | 537.7 | 16.89608 | 545.4 | 22.23846 |
| 30 | 534.2 | 17.77451 | 541.61 | 22.74269 |
| 31 | 528 | 15.95294 | 535.53 | 20.95692 |
| 32 | 517.7 | 10.03137 | 523.77 | 13.49115 |
| 33 | 505.2 | 1.909804 | 509.89 | 3.905385 |
| 34 | 497 | -1.91176 | 500.63 | -1.06038 |
| 35 | 489 | -5.53333 | 494.32 | -3.07615 |
| 36 | 484.3 | -5.8549 | 489.24 | -3.86192 |
| 37 | 480.4 | -5.37647 | 485.3 | -3.50769 |
| 38 | 476.9 | -4.49804 | 481.71 | -2.80346 |
| 39 | 473.5 | -3.51961 | 478.28 | -1.93923 |
| 40 | 470.1 | -2.54118 | 475.25 | -0.675 |
| 41 | 466.8 | -1.46275 | 472.31 | 0.679231 |
| 42 | 468.5 | 4.615686 | 469.94 | 2.603462 |
| 43 | 460.6 | 1.094118 | 467.57 | 4.527692 |
| 44 | 458.2 | 3.072549 | 441.62 | -17.1281 |
| 45 | 456.5 | 5.75098 | 465.58 | 11.12615 |
| 46 | 455.5 | 9.129412 | 463.95 | 13.79038 |
| 47 | 455.1 | 13.10784 | 464.02 | 18.15462 |
| 48 | 454.1 | 16.48627 | 463 | 21.42885 |
| 49 | 452.2 | 18.96471 | 461.61 | 24.33308 |
| 50 | 448.2 | 19.34314 | 459.75 | 26.76731 |
| 51 | 439.5 | 15.02157 | 456.62 | 27.93154 |
| 52 | 420.1 | 0 | 451.47 | 27.07577 |
| 53 | 559.7 | - | 440.94 | 20.84 |
| 54 | 557.5 | - | 569 | - |
| 55 | 554.5 | - | 567.72 | - |
| 56 | 550.1 | - | 565.2 | - |
| 57 | 544.1 | - | 561.62 | - |
| 58 | 535.6 | - | 556.54 | - |
| 59 | 521 | - | 547.2 | - |
| 60 | 505 | - | 528.2 | - |
| 61 | 496.3 | - | 508.33 | - |
| 62 | 491.4 | - | 500.5 | - |
| 63 | 488.6 | - | 496.9 | - |
| 64 | 486.7 | - | 494.7 | - |


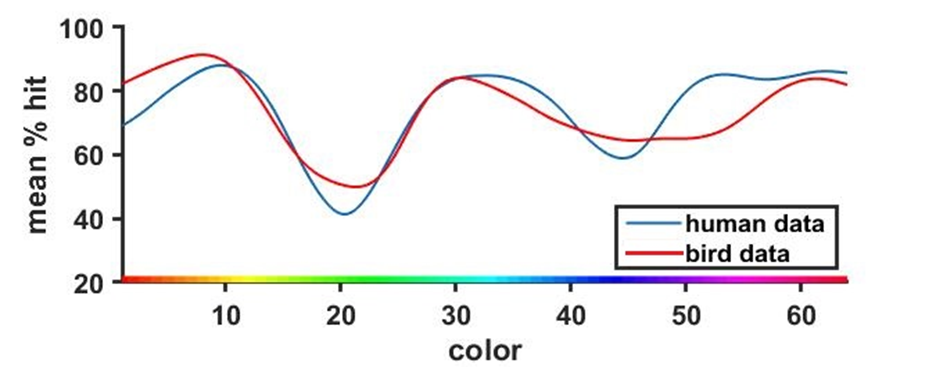


Figure S1: Comparison of the discrimination curves of birds and humans reveals similar patterns in both species. Both show worst performance for colors with green hues. Minima and maxima of both curves overlap. Human results were obtained from a sample of 8 participants with no known abnormalities in color perception. The set-up and paradigm were the same as for the birds.
